# *Mycoplasma gallisepticum* Inactivated by Targeting the Hydrophobic Domain of the Membrane Preserves Surface Lipoproteins and Induces a Strong Immune Response

**DOI:** 10.1371/journal.pone.0120462

**Published:** 2015-03-17

**Authors:** Hazem Atalla, Inna Lysnyansky, Yossef Raviv, Shlomo Rottem

**Affiliations:** 1 Department of Microbiology and Molecular Genetics, The Hebrew University—Hadassah Medical School, Jerusalem, Israel; 2 Division of Avian and Aquatic Diseases, Kimron Veterinary Institute, Beit Dagan, Israel; 3 SAIC-Frederick Inc, National Cancer Institute, Frederick, Maryland, United States of America; Miami University, UNITED STATES

## Abstract

An innovative approach for inactivation of *Mycoplasma gallisepticum* using the hydrophobic photoinduced alkylating probe 1, 5-iodonaphthylazide (INA) is described. Treatment of washed *M*. *gallisepticum* mid-exponential culture (0.2 mg cell protein /mL) with INA followed by irradiation with far-ultraviolet light (310–380 nm) completely abolished viability. Transmission electron microscopy showed that the majority of the inactivated *M*. *gallisepticum* were comparable in size to intact cells, but that part of the INA-treated *M*. *gallisepticum* preparation also contained low density cells and membrane vesicles. Confocal microscopy revealed that untreated *M*. *gallisepticum* cells were internalized by chicken red blood cells (c-RBCs), whereas the INA-inactivated cells remained attached to the outer surface of the c-RBCs. INA treatment of *M*. *gallisepticum* resulted in a complete inactivation of F0F1 –ATPase and of the L-arginine uptake system, but the cytoplasmatic soluble NADH2 dehydrogenase was only partially affected. Western blot analysis of the lipoprotein fraction showed that the INA-treated *M*. *gallisepticum* retained their lipoproteins. Following subcutaneous injection of *M*. *gallisepticum* INA-bacterin, 100% and 68.8% of chickens were positive by the rapid serum agglutination test and enzyme-linked immunosorbent assay respectively, 2 weeks post-injection. These data suggest that the photoinducible alkylating agent INA inactivates *M*. *gallisepticum* but preserves its surface lipoproteins and thus has the potential to be used as a general approach for the inactivation of mycoplasmas for vaccine development.

## Introduction

Mycoplasmas are closely related to Gram-positive bacteria from which they developed by genome reduction [1]. These microorganisms are characterized by a small size (0.2–0.3 μm), minute genome (0.58–1.38 Mb) and the lack of a cell wall and many metabolic pathways [2]. Many species of mycoplasma are known as pathogens and are implicated in a number of serious diseases including atypical pneumonia in man, contagious bovine and caprine pleuropneumonia, contagious agalactia in small ruminants, calf pneumonia, enzootic pneumonia in pigs and chronic respiratory disease in poultry. However, at present there are no effective control measures for many of these infections. Indeed, mycoplasmas are intrinsically resistant to several antimicrobial classes including beta-lactams. Moreover, different species of mycoplasma showed decreased susceptibility to many commercial available antimicrobials (reviewed in [3]). In view of the decreasing efficacy of antibiotics in controlling mycoplasma infections, there is increasing interest in immunoprophylaxis. However, there are few effective vaccines against mycoplasma, most of which provide only transient or partial immunity (reviewed in [4]). Therefore, a new approach is needed in the ongoing pursuit of improved mycoplasma vaccines.

The selective labeling of proteins in biological systems by photosensitization of alkylating probes was previously described [5]. The studies showed that by targeting the hydrophobic domain of enveloped viruses, such as influenza virus, Ebola virus, and a variety of retroviruses, by 1, 5-iodonaphthylazide (INA) followed by photosensitization by irradiation with ultraviolet (UV) light, a complete inactivation of the viruses was achieved while their structural integrity and immunogenicity remained unaffected [6–8]. In this study, we used *Mycoplasma gallisepticum*, the causative agent of chronic respiratory disease in chickens and infectious sinusitis in turkeys [9], as a model to test the potential of photosensitized INA for the inactivation of mycoplasmas for vaccine development.

## Materials and Methods

### Cultivation of *M*. *gallisepticum* and preparation of membranes

*M*. *gallisepticum* strain Rlow (p.8) was obtained from the Mycoplasma Unit Strain Depository at the Kimron Veterinary Institute, Beit Dagan, Israel and was used throughout this study. The organism was grown in a modified Hayflick’s medium [10] supplemented with 10% heat-inactivated fetal calf serum (Biological Industries, Beit Haemek, Israel) at 37°C for 36–48 hrs. Membrane lipids were metabolically labeled by growing the cells in a medium containing 0.3 μCi of [9, 10(N)-^3^H] palmitic acid (53.0 Ci/mmol; New England Nuclear) per mL. In most experiments, the organisms were harvested at the mid-exponential phase of growth (*A*_595_ of 0.12 to 0.14; pH 6.8–7.0) by centrifugation in the cold at 12,000 × *g* for 20 min, washed once and resuspended in a buffer solution containing 0.25 M NaCl and 10 mM Tris-HCl adjusted to pH 7.5 (referred as TN buffer). Paraformaldeyde (PFA) treated *M*. *gallisepticum* cells were obtained as described before [11]. Cell membranes were obtained from washed cells by ultrasonic treatment in a W-350 Heat Systems sonicator operated at 200 W and 50% duty cycles [12] at 4°C for 2 min. The membranes were collected from the cell extracts by centrifugation at 34,000 × g for 30 min, washed once, resuspended in TN buffer, and kept at −70°C until used.

### Treatment of *M*. *gallisepticum* with iodoazidonaphtalenes

Treatment of *M*. *gallisepticum* with iodoazidonaphtalenes was performed as described elsewhere [13, 14]. In brief, the iodoazidonaphtalenes (60–200 μM), INA, 1-azidonaphtalene (AzNAP), 1,5-diazidonaphtalene (DAN), 1,5-diiodonaphtalene (DINAP), and 1-azido 3,5-diiodonaphtalene (DINA) were incubated with washed mid-exponential *M*. *gallisepticum* cells in TN buffer (0.2 mg cell protein /mL) at 37°C for 30 min. Glutathione (20 mmol/L) was then added to neutralize residual iodoazidonaphtalenes in the aqueous phase. The treated samples were irradiated by far-UV light using A 100-W ozone-free mercury arc lamp with a collector lens with a 310 nm cutoff filter (to allow transmission of mercury emission bands of 313, 334, and 365 nm). Irradiation was done at room temperature for 0.5–4.0 min from a distance of 18 cm. The effect of the exposure to iodoazidonaphtalenes and irradiation on the viability of *M*. *gallisepticum* was determined by plating and expressed as colony forming units per mL (CFU/mL). UV-irradiation itself (as described above) was used as a control for the UV- iodoazidonaphtalenes activation.

### Electron and confocal microscopy

For electron microscopy, native or INA-treated *M*. *gallisepticum* cell pellets were fixed with 2% PFA and 2.5% glutaraldehyde (in 0.2 M cacodylate buffer, pH 7.4) for 30 min in the cold. Processing of the samples by osmification, dehydration, and embedding in Epon was performed as previously described [15]. The samples were then sectioned using an LKB-3 ultramicrotome and observed with a Hitachi H-7650 electron microscope equipped with an AMT CCD camera. Confocal microscopy was performed as previously described [16]. In brief, chicken red blood cells (cRBC) were incubated with native, PFA- treated or INA-treated *M*. *gallisepticum* cells at a multiplicity of infection of 10 for 20 hrs at 37°C. The infected cRBC’s were washed twice with phosphate buffered saline (PBS) and fixed with 4% PFA in PBS at room temperature for 10 min. The cells were then permeabilized by incubation with 0.2% Triton X-100 in PBS containing 1% BSA (PBS-BSA solution) for 3 min, washed twice with PBS and blocked for 20 min with 2% horse serum. The cultures were then overlaid for 60 min at room temperature with a fluorescein-labeled burro polyclonal anti- *M*. *gallisepticum* antiserum (NIAID, Bethesda, MD) diluted 1:200 in PBS-BSA solution. The non-bound antibody was removed by rinsing the cover slips three times with PBS. The cover slips were then mounted with a solution containing 90% glycerol, 7% PBS and 3% 1,4-diazabicyclo-(2,2)-octane as an anti-fading agent. The specificity of immunostaining was evaluated by utilizing nonspecific antibodies (non-immune burro serum). Immunofluorescent samples were analyzed using a Zeiss 410 laser scanning confocal microscope (Zeiss, Germany) equipped with an argon ion laser tuned at 488 nm.

### Uptake of L-arginine by *M*. *gallisepticum*

Uptake of ^3^H-L- arginine by *M*. *gallisepticum* was tested in uptake mixtures (total volume of 1 mL) containing washed cells (1 mg of cell protein), 10 mM Tris-MES buffer at various pH values, 250 mM NaCl, 1% bovine serum albumin (fraction V), 10 mM D-glucose and 200 μM L-arginine supplemented with 1.5 μCi ^3^H-L-arginine (L- [2,3,4-^3^H]-arginine-HCl (New England Nuclear, Perkin Elmer Inc.). Chloramphenicol (100 μg/mL) was added to the uptake reaction mixture and the reactions were carried out at 22°C to prevent the incorporation of the labeled arginine into the proteins [15]. The cells were incubated in the uptake mixture for 5 min at 22°C before adding the mixture of the labeled and the unlabeled L-arginine. Incubation was then continued for up to 5 min at 22°C. At various time intervals, 100 μL samples were withdrawn and the reaction was stopped by the addition of 5 mL of ice cold 0.25 M NaCl. The samples were then passed through membrane glass filters (GF/C, 0.45 μm). The filters were washed with 20 mL of 0.25 M NaCl, air-dried, transferred to scintillation vials and counted in a Beckman scintillation counter. Results were presented as DPM per mg cell protein. Efflux of ^3^H-L-arginine from *M*. *gallisepticum* was examined in cells preloaded with the radioactive amino acid. The cells were loaded with ^3^H-L-arginine in the uptake mixture described above for 5 min at 22°C. The cells were harvested by centrifugation and washed and resuspended in the uptake mixture containing various amino acids, except the labeled ^3^H-L-arginine. Samples were withdrawn at various time intervals, filtered, washed, and counted as described above.

### Lipoprotein analysis

The lipoprotein fraction of *M*. *gallisepticum* membranes was obtained by the TX-114 fractionation method [17]. In brief, membranes (1 mg/mL) were incubated in 1 mL TBS containing 1% TX-114 at 4°C for 1 hr with gentle agitation. After centrifugation at 4°C for 30 min in an Eppendorf model 5414 benchtop centrifuge (13,500 × *g*), the supernatant containing the soluble proteins was subjected to three cycles of phase fractionation, including incubation at 37°C for 5 min for micelle formation, followed by centrifugation at room temperature for 3 min at 13,500 × *g* for phase separation, resulting in an upper aqueous phase and a lower detergent phase containing Triton X-114 and lipoproteins. To remove the Triton X-114, the lower phase was treated with Bio-Beads SM-2 Adsorbent (Bio-Rad Laboratories) according to the manufacturer’s instructions. The lipoproteins were kept at −20°C until used.

Lipoproteins were separated by sodium dodecylsulfate-polyacrylamide gel electrophoresis (SDS-PAGE) using 11.5% acrylamide gels followed by Western immunoblot analysis. Separated lipoproteins were visualized by Coomassie Brilliant Blue R-250 stain and electroblotted onto nitrocellulose paper in a Tris-glycine-SDS solution at 250 mA for 2 hr at 10°C. The blotted nitrocellulose paper was then blocked for 1 hr at room temperature with skim milk, incubated for 2 hrs at room temperature with polyclonal chicken anti *M*. *gallisepticum* antibodies (1:500 dilution). To identify the bound immunoglobulins, the immunoblotted nitrocellulose was washed three times with 0.1% Tween 20 in PBS solution and incubated for 1 hr at room temperature with horse reddish peroxidase conjugated rabbit anti-chicken IgG antibodies (Sigma, 1:5000 dilution). The reactive bands were detected using the ECL^TM^ Western blotting detection reagents (Amersham Biosciences) according to the manufacturer’s instructions.

### Analytical methods

Total protein content was determined by Bradford’s method [18] using bovine serum albumin as a standard. ATPase activity of membrane preparations was determined by measuring the release of Pi from ATP [19]. Results were expressed as nmoles Pi released per min/ per mg protein. Reduced nicotine adenine dinucleotide (NADH_2_) dehydrogenase activity in native and INA-treated cells was determined specctroscopically as previously described [20]. All assays were repeated at least three times and were presented as mean ± standard deviation. When there were sufficient time points, the mean of each time was compared using the Wilcoxon Matched-Pairs test.

### Preparation of bacterin

Bacterin was generated from *M*. *gallisepticum* strain Rlow grown for 48 hr in Hayflick’s medium [10] at 37°C. The cultures were harvested, washed, and resuspended in TN buffer and inactivated by treatment with INA (200μM) followed by UV irradiation (MG-INA bacterin), or by 4% paraformaldehyde (MG-PFA bacterin). Each immunization dose contained 1–4 x 10^8^ colony forming units (CFU) as determined after serial platings of *M*. *gallisepticum* cultures prior to inactivation. Freund’s complete adjuvant (Difco, Detroit, MI, USA; 66% by vol.) was then added and the mixture was stirred for 5 min followed by a brief ultrasonic treatment for 1 min at 4°C in a W-350 Heat Systems sonicator operated at 200 W and 50% duty cycles [12]. The obtained bacterins were screened for sterility and stored at 4°C. The negative control was prepared in the same way using sterile TN buffer instead of *M*. *gallisepticum* suspension.

### Assessment of the serological response to MG-INA bacterin

One-day old Lohmann layer breeder male chickens were maintained in negative pressure isolation cells for the duration of the experiment (8 weeks). The Animal Welfare Committee of The Kimron Veterinary Institute approved all animal experiments of this study (Protocol N. b5519). As described in this protocol, the blood samples and cleft swabs were taken from live birds without anesthesia. All efforts were made to minimize suffering during the study and chickens were euthanized by carbon dioxide after the experiments.

At three weeks of age, the birds were bled and the sera checked using the serum-plate-agglutination (RSA) test (SPAFAS, Charles River, USA) to ensure that the chickens were free from *M*. *gallisepticum* and *Mycoplasma synoviae*. At four weeks the birds were divided into three groups: Groups I and II, injected with MG-INA and MG-PFA bacterins, respectively were comprised of 16 chickens each. All birds in Groups I and II were injected subcutaneously midway or lower in the nape of the neck with 0.5 mL of bacterin as follows: subgroups of five to six birds each received bacterin at the concentrations 1×10^8^ CFU/mL, 2×10^8^ CFU/mL and 4×10^8^ CFU/mL, respectively. Group III was a negative control consisting of four birds. Birds in Group III were injected subcutaneously at the neck with 0.5 mL sterile TN buffer, containing 66% (by volume) adjuvant. At 1–4 weeks post-injection (PI), all birds were bled and an antibody response to MG-INA and MG-PFA bacterins was assessed by RSA and enzyme-linked immunosorbent assays (ELISA; SVANOVIR^TM^; Svanova Biotech AB, Sweden) according to the manufacturer’s instructions. In addition, at 2–3 weeks PI, cleft swabs were taken from chickens in all groups for DNA extraction and PCR testing to ensure that they were free of *M*. *gallisepticum*. Genomic DNA was extracted using the Maxwell DNA Isolation Kit for Cell/Tissue and the Maxwell 16 apparatus (Promega) according to the manufacturer's instructions and subjected to PCR testing with diagnostic primers complementary to the *mgc2* gene as previously described [21].

## Results

A variety of UV-activatable azido and iodo-based hydrophobic compounds were tested for their ability to inactivate the wall-less bacterium *M*. *gallisepticum*. To assess the efficacy of treatment with iodoazidonaphtalenes on the viability of the *M*. *gallisepticum* strain Rlow, we tested three parameters: (i), the residual viability of *M*. *gallisepticum* after the treatment; (ii), the structural and functional integrity of *M*. *gallisepticum* after inactivation; and (iii) the ability of the treated *M*. *gallisepticum* cells to elicit a humoral response in chickens.

### The effect of UV and iodoazidonaphtalenes on the viability of *M*. *gallisepticum*

**Fig 1:**
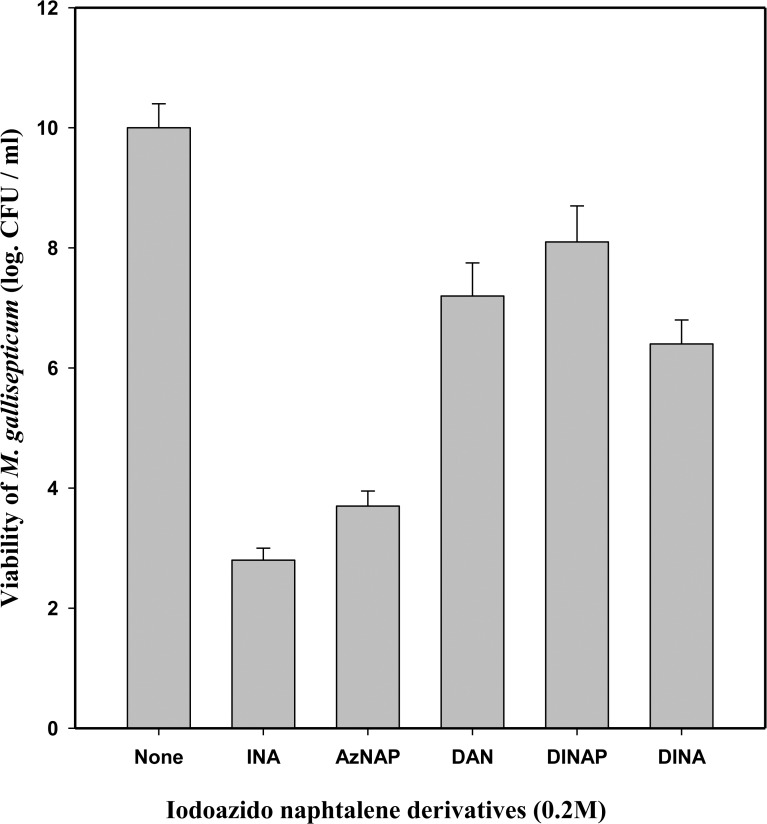
The effect of treatment with iodoazidonaphtalenes followed by UV photoinduction on the viability of *M*. *gallisepticum*. *M*. *gallisepticum* Rlow log-phase (exponential) culture were harvested, washed twice and resuspended in TN buffer to a final concentration of 0.2 mg/mL cell protein. The cells were treated with 200μM 1, 5-iodonaphthylazide (INA), 1-azidonaphtalene (AzNAP), 1,5-diazidonaphtalene (DAN), 1,5-diiodonaphtalene (DINAP) and 1-azido 3,5-diiodonaphtalene (DINA) at room temperature, irradiated by UV for 2 min and viability was determined as described in Materials and Methods.

To begin to explore the effect of UV-irradiation of *M*. *gallisepticum* cells treated with iodoazidonaphtalenes, we tested the effect of UV-irradiation (a control for the UV-iodoazidonaphtalenes activation) on the viability of *M*. *gallisepticum* (Table 1). The cells were irradiated at room temperature as described in Materials and Methods. Irradiation of up to 2 min had very little effect on *M*. *gallisepticum* viability, whereas a decrease of 2–3 logs in CFU/mL was measured after 4 min of irradiation. In continuation, we tested the effect of the iodoazidonaphthalene derivatives on the viability of *M*. *gallisepticum* (Fig. 1). The results showed that treatment with INA or AzNAP followed by UV irradiation for 2 min from a distance of 18 cm decreased the viability of *M*. *gallisepticum* by 6–8 logs CFU/mL. In contrast, a treatment with DINA, DAN, or DINAP had a little effect (Fig. 1).

**Table 1:** The effect of UV irradiation on the viability of *M*. *gallisepticum*.

Irradiation time (min)	Viability (CFU / ml)
0	9.0 ± 2.4 x 10^9^
0.5	7.2 ± 3.6 x 10^9^
1.0	5.7 ± 2.1 x 10^9^
2.0	6.3 ± 1.2 x 10^9^
4.0	1.2 ± 0.8 x 10^7^

**Fig 2:**
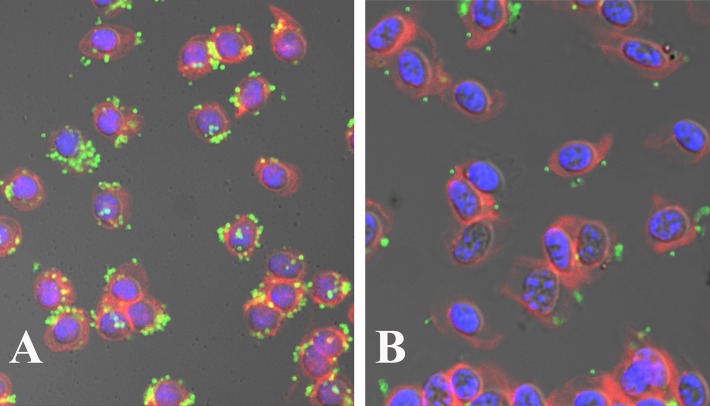
Confocal micrographs depicting the interaction of INA treated *M*. *gallisepticum* with cRBC. cRBS were infected at a MOI of 100 with untreated *M*. *gallisepticum* cells (A) or with *M*. *gallisepticum* cells treated with INA followed by UV irradiation (B).

In continuation, to test the lack of viability of *M*. *gallisepticum*-INA treated cells, we checked their ability to invade cRBCs. The cRBCs were infected by untreated and INA-treated *M*. *gallisepticum* as described in Materials and Methods. Following infection of cRBCs by untreated *M*. *gallisepticum*, the surface and intracellular foci of fluorescence, corresponding to extracellular and intracellular mycoplasmas, were seen (Fig. 2A). The amounts of intracellular *M*. *gallisepticum* increased during the first 24 h of infection (data not shown). Invasion was temperature dependent; thus, at 4°C, fluorescence was predominantly associated with the cell surface of the host cells (data not shown). However, there was no evidence for invasion following infection with INA treated *M*. *gallisepticum* (Fig. 2B) or with PFA-treated *M*. *gallisepticum* (data not shown), and only numerous extracellular foci of fluorescence, corresponding to surface-bound mycoplasma, were seen.

### The effect of INA treatment on the structural and functional integrity of *M*. *gallisepticum*

**Fig 3:**
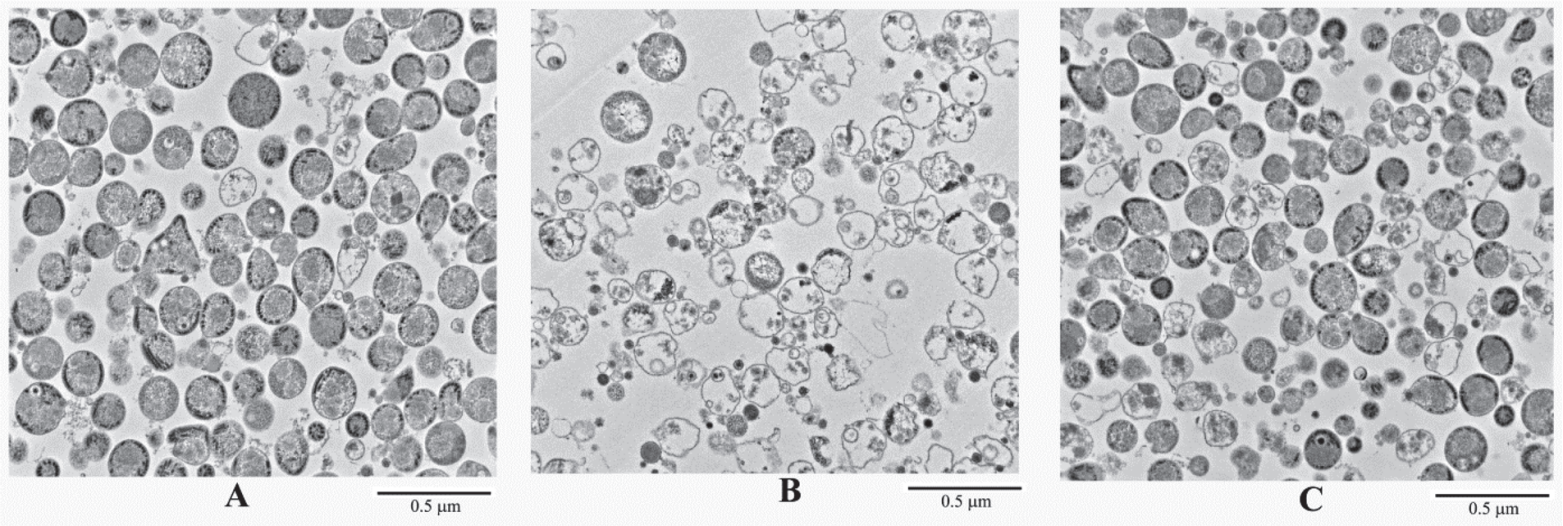
Transmission electron micrographs of INA treated *M*. *gallisepticum*. Washed *M*. *gallisepticum* Rlow preparations were treated with 200 μM INA, irradiated by UV light and analyzed by transmission electron microscopy as described in Materials and methods. (A), untreated *M*. *gallisepticum*; (B), *M*. *gallisepticum* treated with INA at 37°C; (C), *M*. *gallisepticum* treated with INA at 4°C.

To examine possible changes in structural integrity of *M*. *gallisepticum* cells as a result of INA treatment, thin sections of untreated and treated *M*. *gallisepticum* cells samples were examined using transmission electron microscopy. Fig. 3 shows that after treatment with 200 μM INA for 30 min at 37°C followed by irradiation, *M*. *gallisepticum* cells appear to swell and lyse, resulting in cells with a relatively low cytoplasmic content (Fig. 3B) in comparison with untreated cells (Fig. 3A). Swelling and subsequent lysis of the INA-treated *M*. *gallisepticum* cells was significantly lower when the INA treatment was performed at 4°C (Fig. 3C). The total protein content of the treated cells was lower (65–79%) than that of the untreated cells. However, the lipid content, determined by measuring of radioactivity of metabolically labeled lipids, obtained from the cells growing in a medium containing [^3^H]-palmitic acid, was almost the same (data not shown).

**Fig 4:**
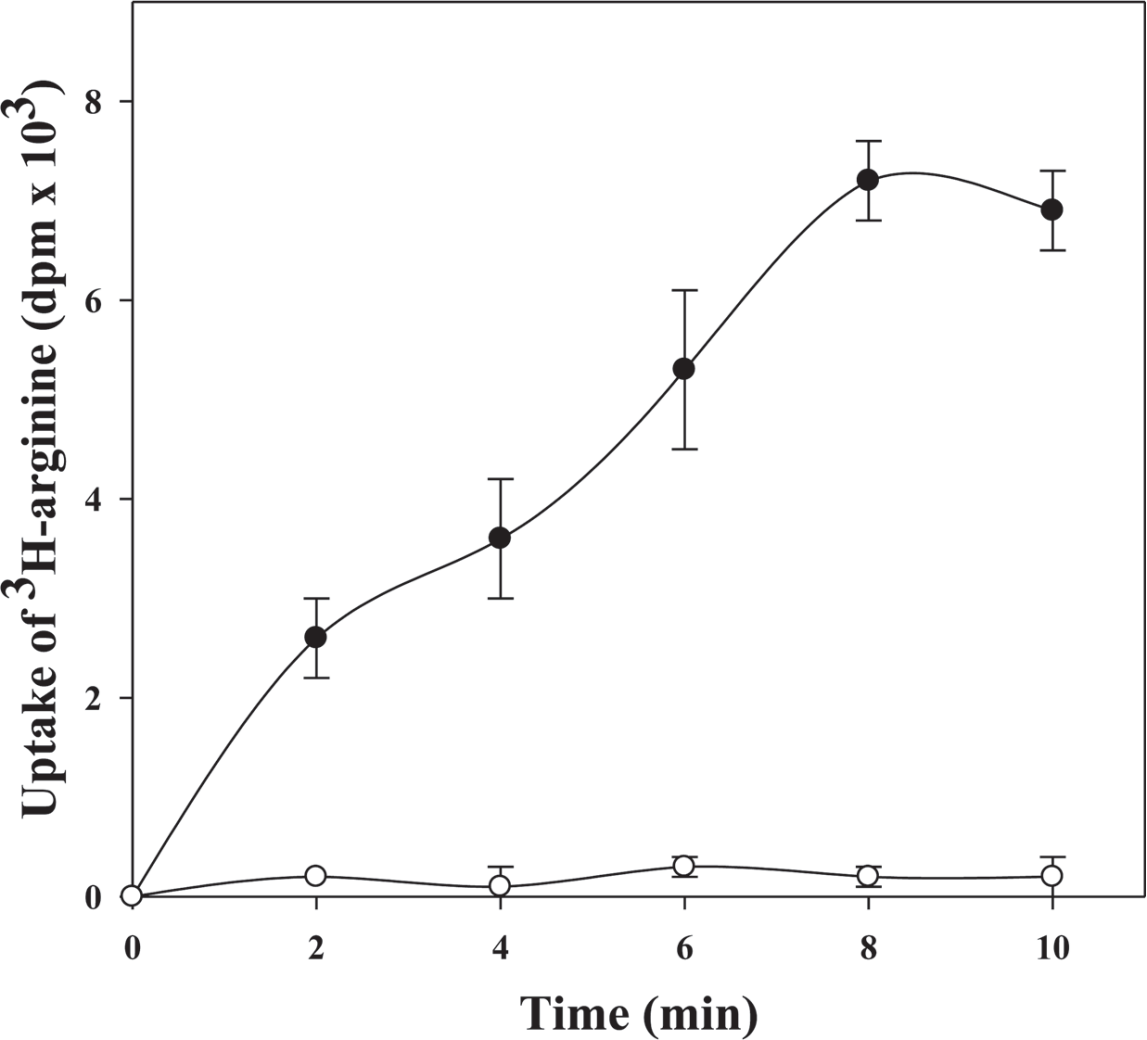
The effect of INA treatment on ^3^H-*L*-arginine uptake by *M*. *gallisepticum*. *M*. *gallisepticum* Rlow cells (0.2 mg cell protein/mL) were treated with INA followed by UV irradiation and tested for ^3^H-*L*-arginine uptake. Open symbols, INA treated cells; closed symbols, control of untreated cells.

The functional integrity was obtained by analyzing representative membrane-bound enzymatic activity and the arginine transport system. Table 2 shows that INA treatment of *M*. *gallisepticum* resulted in a complete inactivation of the membrane-bound F_0_F_1_ –ATPase, but that the cytoplasmatic soluble NADH_2_ dehydrogenase was only partially affected. Both, NADH_2_ dehydrogenase and F_0_F_1_ –ATPase were completely inactivated after PFA treatment (Table 2). To evaluate the effect of INA treatment on *M*. *gallisepticum* transport activities, the uptake of L-arginine by untreated and INA-treated *M*. *gallisepticum* was determined. Although the recommended growth temperature of *M*. *gallisepticum* is 37°C, our uptake experiments were performed at 22°C where only a negligible portion of the L-arginine taken up was incorporated into macromolecules (data not shown). Fig. 4 shows the uptake of L-arginine as a function of time. While the L-arginine uptake system was active in untreated *M*. *gallisepticum* cells; no uptake activity was detected in the INA treated cells.

**Table 2:** The effect of INA treatment of *M*. *gallisepticum* cells on ATPase and NADH_2_ dehydrogenase activities.

*M*. *gallisepticum*	ATPase activity*	NADH_2_ dehydrogenase activity**
Untreated	3.35 ± 0.27	1.67 ± 0.30
INA treated	0.37 ± 0.04	0.98 ± 0.25
PFA treated	0.03>	0.02>

*M*. *gallisepticum* strain R low cells grown in Hayflick’s medium were harvested, washed and treated with INA followed by UV irradiation. Cells treated with 4% paraformaldehyde (PFA) served as a negative control. The treated cells were disrupted by ultrasonic treatment [12] and ATPase and NADH_2_ dehydrogenase activities were determined in the cell extract as described in Materials and Methods. The results are presented as specific activities and are means ± standard deviation of three independent experiments.

*Specific activity expressed as μmoles of inorganic phosphorous released per mg cell protein per 30 min.

**Specific activity expressed as the decrease in absorbency at 340 nm per mg cell protein per min.

### The effect of INA treatment on membrane-protein and lipoprotein profiles

**Fig 5:**
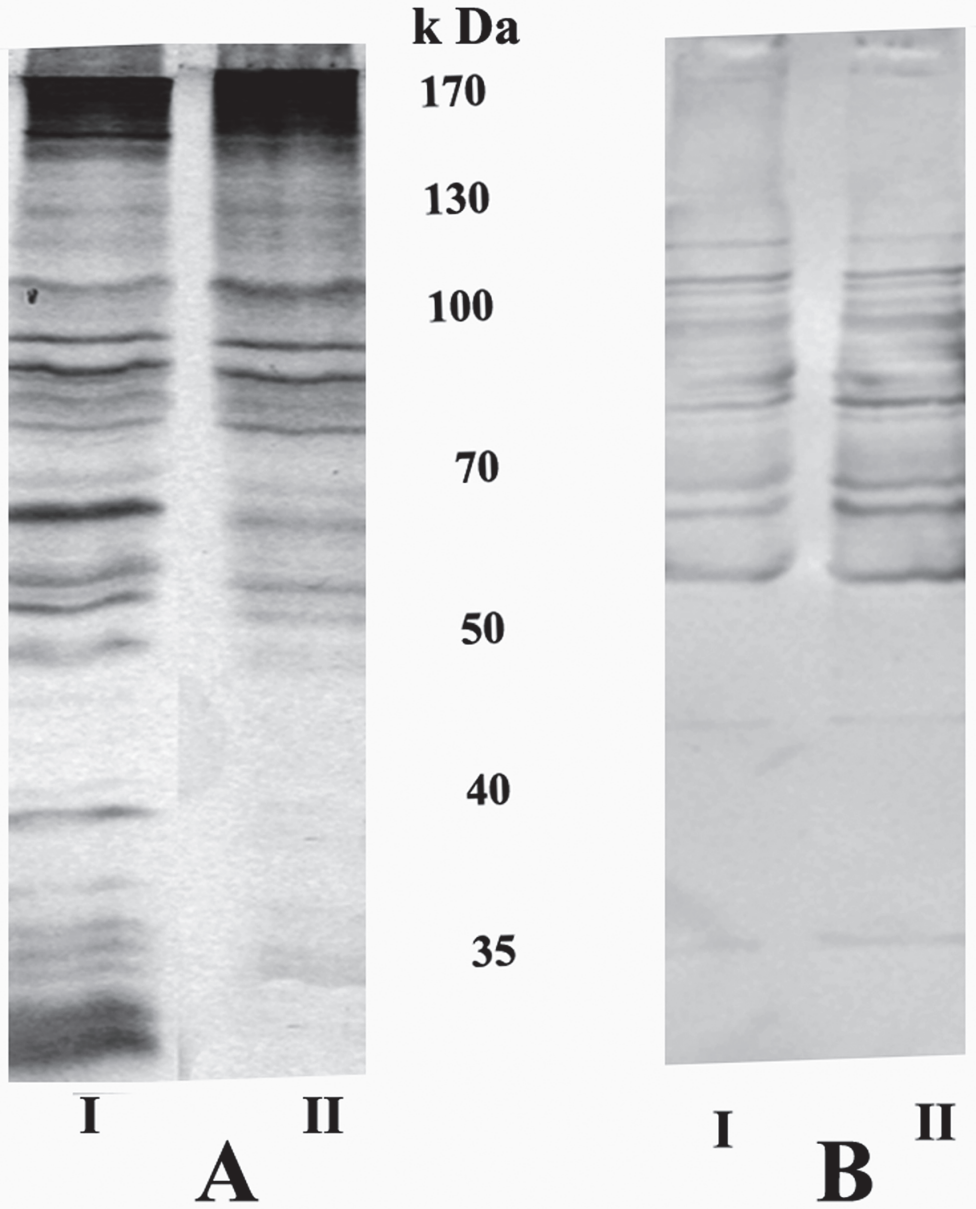
The effect of INA treatment on the membrane protein and on the lipoprotein profiles of *M*. *gallisepticum* Rlow. Western immunoblot analysis of isolated *M*. *gallisepticum* membranes (A) and the lipoprotein fraction (B) obtained from untreated (I) or INA treated cells (II).

When membrane preparations from untreated and INA treated *M*. *gallisepticum* were separated using SDS-PAGE, the electrophoretic mobility of the major high molecular weight bands (>50 k Da) of *M*. *gallisepticum* membrane proteins did not change, but among lower molecular weight bands (<50 kDa) some bands were missing and the intensity of other bands was much lower in the INA-treated than in the untreated preparations (Fig. 5A). Western immunoblotting of the lipoprotein fraction of untreated and INA-treated *M*. *gallisepticum* cells revealed that the pattern and intensity of the lipoprotein bands were almost the same in both (Fig. 5B).

### Systemic antibody response to INA treated *M*. *gallisepticum*

The antibody response of chickens immunized with the INA-treated *M*. *gallisepticum* as evaluated by RSA and ELISA assays at 1–4 weeks after injection of the bacterins is presented in Table 3. No antibodies against *M*. *gallisepticum* and *M*. *synoviae* were detected using RSA in the serum of any bird (n = 36) prior to injection. No antibody response against *M*. *gallisepticum* was detected in non-injected chickens (Group III) during the trial. At 1 wk PI, serological activity was detected by RSA in 6.25% and 12.5% of INA and PFA-injected birds, respectively, and by ELISA at 2 wks PI (68.8% and 88.8%, respectively). All the birds in both groups were positive by 2 weeks PI (RSA) and 3 weeks PI (ELISA). There were no significant differences between sub-groups injected with different concentrations of *M*. *gallisepticum* bacterin (see Materials and Methods; data not shown). DNA extracted from the cleft swabs taken from the chickens in all groups at 2–4 weeks PI was negative in the *mgc2*-PCR test (data not shown).

**Table 3:** Serological response by chickens injected with MG-INA and MG-PFA bacterins.

Group	Injected with	Week 1		Week 2		Week 3	
		% RSA^+^	% ELISA^+^	% RSA^+^	% ELISA^+^	% RSA^+^	% ELISA^+^
I	MG-INA	6.25 (16)	NT	100 (16)	68.8 (16)*	100(16)	100 (16)
II	MG-PFA	12.5 (16)	NT	100 (16)	88.8 (16)**	100 (16)	100 (16)
III	uninjected	0 (4)	NT	0 (4)	0 (4)	0 (4)	0 (4)

Total number of chickens in each group is shown in parentheses.

NT—not tested.

* In addition, the 18.8% (3/16) serum samples were categorized as doubtful according to the manufacturer’s instructions (ELISA; SVANOVIR^TM^; Svanova Biotech AB, Sweden).

** In addition, the 11.2% (2/16) serum samples were categorized as doubtful according to the manufacturer’s instructions (ELISA; SVANOVIR^TM^; Svanova Biotech AB, Sweden).

The % of seropositive chickens, tested by RSA and ELISA at 4 weeks PI, was the same as at 3weeks PI (data not shown).

## Discussion

In this study, we examined the concept that the lipid bilayer of the membrane can be used as a multicomponent target for inactivation of mycoplasmas for vaccine application. Iodoazidonaphtalenes have been applied because these hydrophobic compounds were originally developed to partition into the lipid domain of biological membranes and selectively bind upon irradiation by far-UV light to the transmembrane domains of integral membrane proteins [5, 6]. Among the various hydrophobic alkylating probes tested in our study, INA was found to be most effective in decreasing the viability of *M*. *gallisepticum* by about 7 logs CFU/mL (Fig. 1). Moreover, confocal microscopic studies showed no invasion to the cRBC by *M*. *gallisepticum* cells treated with INA compared to untreated cell (Fig. 2) and pointed out to complete abolishment of its viability. *M*. *gallisepticum* was regarded as a non-invasive surface parasite [22] that colonizes epithelial surfaces of chicken tissues by interactions between *M*. *gallisepticum* specific cytadhesins and their corresponding host cell receptors [23]. Nonetheless, studies using the gentamicin invasion assay and a double immunofluorescence microscopic technique indicate that viable *M*. *gallisepticum* strains Rlow and Rhigh cells are capable of invading non-phagocytic host cells and surviving within them [24, 25].

The transmission electron microscopy results, obtained in this study, showed that most of the INA-treated *M*. *gallisepticum* were comparable in size to untreated cells, but low-density cells and membrane vesicles were also obtained (Fig. 3). This partial lysis is apparently due to the inhibition of the membrane bound ATPase activity of the INA-treated *M*. *gallisepticum* cells as shown in Table 2. As mycoplasmas lack a rigid cell wall, a major goal of these organisms is to maintain osmotic stability. This is achieved by the active pumping out of small molecules, such as Na^+^ ions that permeate into the cells due to the disordered boundaries at the interface between the fluid and solid regions within the mycoplasmal membrane lipid bilayer [26]. The pumping out of Na^+^ ions is achieved by an energy-dependent process operated via the membrane proton translocated ATPase and a Na^+^-H^+^ exchange carrier. Indeed, dicyclohexylcarbodiimide, a specific inhibitor of proton translocated ATPases was previously shown to induce cell swelling and partial lysis of *M*. *gallisepticum* [26].

Our results that *M*. *gallisepticum* retained their surface lipoproteins after INA treatment (Fig. 5) makes sense as INA partitions into the lipid domain, causing the specific inactivation of integral membrane proteins embedded in this domain, while maintaining the integrity and activity of proteins that protrude outside the membrane, such as lipopoproteins [6]. Lipoproteins are extremely abundant in the cell membrane of mycoplasmas. All membrane anchored bacterial lipoproteins contain a lipoylated amino-terminal cysteinyl residue which, in some cases, is *N*-acylated. Chemical analyses of mycoplasmal lipoproteins have revealed that their lipoylation mechanism is similar to that of Gram-negative and Gram-positive bacteria [22]. However, in most mycoplasmas the lipoproteins are not *N*-acylated (Fig. 3), nor has an N-acyltransferase gene been found in the genome [27].

Mycoplasmal lipoproteins are pro-inflammatory eliciting both innate and adaptive immune responses. Moreover, the lipoproteins are among the most dominant immunogens in mycoplasmas which have a role in virulence-associated functions, such as colonization, invasion, and evasion of host defense [2, 28]. For example, initial analysis of the genome sequence of *M*. *gallisepticum* Rlow identified 80 putative lipoproteins [29]. Among them the most abundant is the *vlh*A gene family consisting of 51 genes [30], which encode immunodominant lipoproteins and hemagglutinins that undergo phase-variable expression both in vitro and in vivo [31, 32]. The *vlh*A genes facilitate establishment of chronic infection through immune evasion and thus are thought to be important pathogenicity factors. In view of the aforementioned, preservation of surface lipoproteins after INA treatment is of interest and requires future investigations in order to understand if the resultant bacterin will elicit protection against challenge.

It has been shown that INA treatment of enveloped viruses results in preservation of surface epitopes, which is critical for an effective immune response [7, 8]. Indeed, our results demonstrated that INA-treated *M*. *gallisepticum* elicited a strong humoral immune response (Table 3). Despite the fact that PFA-treated *M*. *gallisepticum* also elicited a strong antibody response (Table 3), the crosslinking of proteins by PFA has been shown to distort the structure of immunogenic epitopes [33–35]. Indeed, the method of inactivating bacteria to develop bacterin has a major influence on the outcome of immunization. It is crucial that the substances used for inactivation cause minimal alteration of the antigenes. For example, vaccination of ewes with phenol- or saponin-inactivated *Mycoplasma agalactiae*, but not with formalin, sodium hypochlorite or heat-inactivated *M*. *agalactiae*, elicited high antibody levels and resulted in protection to experimental challenge [33]. Overall, the efficacy of inactivated vaccines for protection against disease caused by pathogenic mycoplasmas is controversial. In swine production, vaccination with bacterins (alone or in combination with antibiotics) is frequently used worldwide to control *M*. *hyopneumoniae* disease [36] and it is still considered the most effective practice [37]. Many efforts are being directed toward the developing of new vaccines that may offer a better protection against *M*. *hyopneumoniae* infections, including recombinant subunit vaccines and DNA vaccines (reviewed by [38]). However, none of the experimental vaccines are currently able to offer total protection or a similar protection as the commercial vaccines [38]. In poultry, commercial inactivated *M*. *gallisepticum* vaccines have been used for control of disease in several countries despite the fact that they do not prevent infection. Although, there are some reports showing that *M*. *gallisepticum* bacterins do protect chickens against respiratory signs, airsaculitis, egg production losses and reducing egg transmission (reviewed by [39]). The major advantage of bacterins vis à vis live attenuated vaccines is their safety. Live attenuated vaccines may have residual pathogenicity or may revert to the status before attenuation as has been reported, for example, for *M*. *gallisepticum* 6/85 and ts-11 vaccine strains [40–42].

## Conclusions

In general terms, this study demonstrates that hydrophobic alkylating compounds, such as INA, completely inactivate *M*. *gallisepticum* with the preservation of its surface lipoproteins. The chicken immunization experiments show that INA-inactivated *M*. *gallisepticum* induces a robust serum antibody response in chickens and suggest that INA has a potential as a new inactivating agent for use in vaccine formulation. However, further analysis of INA-inactivated *M*. *gallisepticum* in the animal model will be needed to test the protective efficacy of obtained INA-bacterin.
